# Thinking of Oneself as Someone: The Structure of Self‐Representation

**DOI:** 10.1111/cogs.70186

**Published:** 2026-02-23

**Authors:** Julian Hauser

**Affiliations:** ^1^ Facultat de Filosofia, LOGOS Universitat de Barcelona

**Keywords:** Allocentric representation, Animal cognition, Egocentric representation, Infants, Representational format, Self‐representation

## Abstract

One question we can ask when investigating the nature of self‐representation concerns the types of property that must figure in its content. Here, authors have claimed that self‐representations must be about spatial, temporal, bodily, or mental properties. However, we can also ask a second question: *how* do we need to represent a property to *self*‐represent it? I address this latter question. I argue that a distinction between egocentric and allocentric forms of representation—known from spatial cognition—also applies to representations of other kinds of property. I use examples drawn from animal cognition and developmental psychology to show how creatures allocentrically represent their temporal, bodily, and cognitive properties. These representations are minimal self‐representations: they represent one's properties so that an explicit differentiation is made between the system and other objects (or between the system's actual and merely possible properties), they are directly linked to behavior and sensation, and they are immune to error through misidentification. The upshot is a view on which different creatures may self‐represent more or fewer kinds of property. More substantive forms of self‐representation (for instance, as exemplified by neurotypical adult human beings) then require integrated minimal self‐representations of the right kinds of property.

Representations may be about different kinds of property, and they may represent these properties in different ways. A red apple may be represented by an image of a red apple or the words “red apple.” The matter is no different for mental representations: I can token the phrase “red apple” in inner speech or conjure up an image of a red apple. This paper concerns the representations with which a system represents its own properties. I argue that these representations are (minimal) *self*‐representations when they represent properties with *coordinated allocentric representations*. Self‐representation does not depend on *what* properties are represented but only on *how* they are represented.

A minimal self‐representation is the simplest kind of representation that is a genuine self‐representation. A genuine self‐representation has *de se* content; with it, a system represents *itself qua itself*. To do so, the representation must fulfill two conditions. First, it must explicitly differentiate between the system's properties and those of other objects. A creature that represents a *tree to be located to the left* does not self‐represent, since the fact that the object is to the left *of the creature* is not made explicit. Such a representation merely *concerns* the self, whereas a self‐representation is *about* it (Perry, 1993). Second, the creature must be disposed to employ the representation in a way that manifests its knowledge that it represents itself. An animal fails to self‐represent when it represents the animal it sees in the mirror without recognizing that it is that animal. The knowledge required here is a kind of knowledge‐how (Ryle, 2009): the creature must be disposed to update the representation based on sensation and employ the representation to guide its behavior (Evans, 1982).

I argue that minimal self‐representations are coordinated allocentric representations. We know allocentric representations from the literature on spatial cognition, where they appear in the guise of cognitive maps (O'Keefe & Nadel, 1978; Rescorla, 2017). A fruit bat, for instance, may represent its own and a fruit tree's location with two representational tokens that imply their spatial relation (Tsoar et al., 2011). Such a representation *explicitly* differentiates between the bat's and the fruit tree's location. This representation is *about* the bat.

Allocentric representations contrast with egocentric representations, such as those employed in path integration. Such representations do not contain a representational token standing for the representer—they only encode some relation to a target state. Egocentric representations, hence, only concern the self (and are not about it). Egocentric representations necessarily have sensorimotor implications: if an ant represents a nest to be located such‐and‐such a distance to its left, then this implies that it would reach the nest were it to engage in certain movements.

Allocentric representations, in contrast, require *coordination* to be useful. Otherwise, such a representation may not represent the system at all (think of a bat that does not know where it is), or it may represent the system in such a way that the system does not know this is the case (think of the above mirror case). Coordination requires a special representational token, a self‐token, that the bat takes to refer to itself. This gives the bat the know‐how to use its cognitive map by establishing a two‐way information flow from the allocentric representation to sensorimotor information (which is in an egocentric format). The bat can now use its representation to guide its behavior, and it can update the representation based on sensations. The bat now instantiates a self‐representation.

A central argument in this paper is that coordinated allocentric representations can be used to represent many kinds of properties besides spatial ones. I discuss evidence for allocentric representations of temporal, bodily, and cognitive properties in a range of human and nonhuman animals. For instance, a forward model (Grush, 2004; Wolpert & Ghahramani, 2000) implies an explicit differentiation between possible bodily states, making it an allocentric representation. To predict a future bodily state, the model needs to be supplied with the system's actual state, making it a coordinated allocentric representation.

This brings me to my main claim: whenever a system represents *any* property with a coordinated allocentric representation, it instantiates a minimal self‐representation. According to this structural account, self‐representation is determined only by whether a system represents its properties with the right sort of cognitive architecture.

This proposal rests on a philosophically rigorous treatment of a cognitive architecture that researchers can readily operationalize in empirical studies. The approach thus bridges theoretical sophistication with practical application, fostering collaboration across the cognitive sciences. For empirically minded researchers, I offer an account of self‐representation that can be applied by extending paradigms from spatial cognition (and beyond) to study self‐representation in new domains. The account enables comparative studies that illuminate commonalities and distinctions in how different agents—human and nonhuman animals and potentially even artificial systems—develop and deploy self‐representations. Such research promises insights into the evolutionary origins and ontogenetic trajectories of self‐representation.

Philosophically oriented researchers will discover a rich territory for extending investigations beyond the linguistically capable adult human beings that still dominate the literature on self‐representation. The account's structural nature provides a unifying framework for examining specific representational formats, including mental imagery, iconic representation, and cognitive maps. The account also challenges views that insist self‐representations must contain specific types of content—such as representations of others’ mental states (e.g., Musholt, 2012).

The paper begins with four sections examining coordinated allocentric representations across different creatures, starting with a section on spatial representations that introduces key concepts. These sections imply a link between coordinated allocentric representation and self‐representation. Section 5 argues that coordinated allocentric representations are genuine self‐representations, while Section 6 looks at how creatures infer their properties. Section 7 looks at how an account of minimal self‐representation can help us study the emergence of more complex forms of self‐representation. Section 8 concludes.

## 1 Spatial cognition and minimal self‐representation

The distinction between egocentric and allocentric representation arose in the literature on spatial cognition, and it is here that some (e.g., Grush, 2000) have first suggested links to self‐representation. Spatial cognition also provides the most intuitive domain in which to introduce the core ideas developed in later sections—all the more reason to start here.

The *Cataglyphis* desert ant has become a poster child for path integration (C. R. Gallistel, 1989; Reid, Latty, Dussutour, & Beekman, 2012). These ants can return home from long foraging expeditions despite their featureless desert habitat. Moreover, if we capture a homeward‐bound ant and transfer it to a different location, it will proceed in the direction where the nest would have been, had we not relocated it (Wehner & Srinivasan, 1981).

In path integration, a creature encodes objects’ locations through 〈*distance, bearing*〉 tuples specifying relations from the current location. For instance, a desert ant may represent its nest to be 200 steps in a certain direction. As it moves, the ant must update its representation to keep track of the object, which it does using proprioceptive information about steps taken (Wittlinger, Wehner, & Wolf, 2006). This explains how the ant can successfully navigate without external cues and why displaced ants continue in the original direction when proprioceptive inputs are absent.

**Fig. 1:**
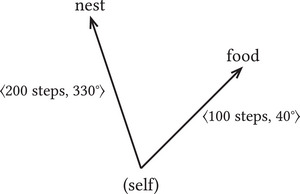
Egocentric representation.

Path integration employs *egocentric* spatial representations where the origin of the represented relations is the representer itself (Grush, 2000). A 〈*distance, bearing*〉 tuple represents spatial relations *from the representer's current location*. A creature employing such a representation arranges objects around itself. The ant may represent its nest to be *200 steps away at an angle of 330°*, whereas the food source is a *100 steps away at an angle of 40°* (see Fig. 1).^1^ Egocentric representations are vectors that represent relations between the system's actual state and target states.

An egocentric representation of spatial properties contains tokens standing for the distance and bearing. We, therefore, say that these properties are *explicitly* represented. Something is explicitly represented “if and only if there actually exists in the functionally relevant place in the system a physically structured object […] for which there is a semantics or interpretation, and a provision (a mechanism of some sort) for reading or parsing the formula” (Dennett, 1982, p. 216). The ant's representation fulfills this condition: it contains a 〈*distance, bearing*〉 token, and (presumably) there exists a mechanism with which the ant can parse this token.

In contrast, the fact that the 〈*distance, bearing*〉 token tracks *a relation between the system's location (and orientation)* and some object is only *tacitly* represented. The representation merely *concerns* the system rather than being *about* it (Perry, 1993). The ant uses the representation to navigate without explicitly representing that representational tokens relate to itself—it simply employs *all* tokens as representing such relations, using them to compute how it (and only it) can get to places.^2^ In other words, an egocentric representation explicitly represents a vector whose initial point is not specified, but tacitly, that point is given by the representer's actual state.

The same conclusion emerges when we consider what makes the ant's representation accurate. Intuitively, if the nest is not located where the ant represents it to be, then the representation is inaccurate. Yet to evaluate this claim, we need more information than the representation's explicit content provides. The ant's location, after all, forms no part of that explicit content, but we require this detail to assess the egocentric representation's accuracy. This explains why philosophers describe the representer as an *unarticulated constituent* of the representation (Perry & Blackburn, 1986). Though absent from the representation's explicit content, the representer must nonetheless be invoked when we assess the representation's accuracy.

As the representations employed in path integration do not contain a token standing for the system, they cannot underwrite an explicit differentiation between self and other. These representations concern but are not about the self, making them strictly speaking selfless (David Lewis, 1979; see also Recanati, 2024) and, hence, not genuine self‐representations.

To find an instance of genuine self‐representation, we need to look toward creatures whose more sophisticated behavioral profile relies on allocentric representation. In a recent experiment, Tsoar et al. (2011) captured fruit bats in their cave and released them in, or just outside, a crater about 80 km away. The bats that found themselves within the crater, and unable to sense any of their home range's landmarks, engaged in random exploratory flights around the crater. Only after clearing the crater's cliffs did they head home. Bats released outside the crater flew home immediately.

**Fig. 2:**
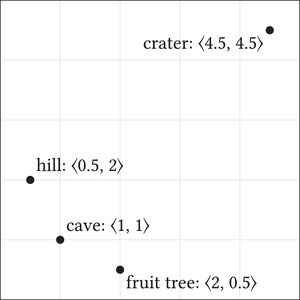
Allocentric representation.

Tsoar et al. (2011) take the experiment to show that bats use *cognitive maps* (see O'Keefe & Nadel, 1978; Rescorla, 2017) to navigate their environment. We may think of these as representing spatial relations in a manner akin to our phones’ navigation apps, with various tokens standing for the represented objects and the relations between tokens giving their spatial relations (see Fig. 2).^3^ Importantly, they allow the system to represent spatial relations between nonself objects. This enableas behavioral abilities unattainable with egocentric spatial representations.

With a cognitive map, a bat can plot novel routes to goal locations despite not being able to perceive them. We could see evidence for this in the bats’ behavior: after determining distances and bearings to landmarks like hills and settlement lights, they computed direct routes home. Note that the experiment carefully excluded alternative explanations: the crater was outside their home range (ruling out associative links), they were transported (excluding path integration), and the design controlled for magnetic, olfactory, and celestial navigation.

Cognitive maps are *allocentric* spatial representations (Grush, 2000). Allocentric representations contain tokens with which they explicitly represent certain objects (for instance, the cave and a distant hill). Note how these tokens do not, unlike in the egocentric case, tacitly refer to the location of the representer itself. An allocentric representation can represent spatial relations as, in principle, independent of the system's location: the system's location does not need to figure in the representation. And when the system's location does not figure in the representation, then locomotion does not entail the need for accuracy‐preserving updates.

To navigate with an allocentric representation, you need to know where you are. The bat, to find its way home after being released, must first exit the crater and figure out how its own location relates to some of the represented objects. It must enrich its map with a representational token that stands for itself, explicitly differentiating between its own and other objects’ locations. This representation is *about* the self.

However, genuine self‐representation requires more than just a token that happens to be about oneself—the system must also have knowledge‐how to the effect that this token refers to itself. Consider a bat seeing itself in a mirror but failing to recognize its reflection. Although the resulting representation includes a token that refers to the bat, the bat cannot use this information to guide its behavior. Following the literature on *de se* thought (Castañeda, 1966; Perry, 1979), I distinguish between mere self‐tokens (that just happen to be about oneself) and self*‐tokens (which imply knowledge of the fact that they are about oneself). Only self*‐tokens bring about genuine self‐representation with *de se* content. To keep the terminology simple, I will use “self‐token” to refer to self*‐tokens, except when the distinction becomes relevant again in later sections.

To possess knowledge‐how to the effect that a self‐token is about oneself means being disposed to use that token in ways that connect it directly to sensorimotor information. Such information maps motor outputs to sensory inputs, allowing a system to predict what it will sense as a result of particular movements—and thus to act toward desired sensory outcomes. Because sensorimotor information invariably relates the *representer*’*s own* motor outputs to sensory inputs, it is fundamentally egocentric. We can think of sensorimotor representation as a vector: its magnitude specifies required motor outputs, while its endpoint specifies the goal state in terms of expected sensory input. When a system deploys a representation containing a self‐token, it becomes disposed to translate between such sensorimotor information and allocentrically represented information.

**Fig. 3:**
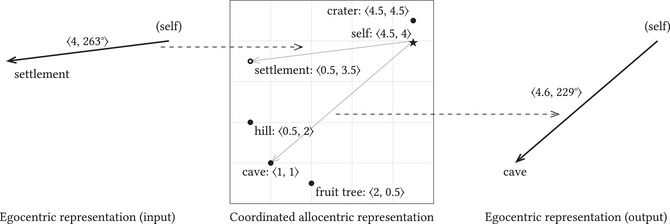
Coordination between allocentric representation and sensorimotor information.

Consider how this works in allocentric spatial representation (see Fig. 3). Here, sensorimotor information can be derived from the vector in the allocentric representation that originates at the self‐token and terminates at the token marking the goal location. Take a bat that allocentrically represents itself as being a certain distance from its cave. From this spatial relationship, the bat can infer a sensorimotor representation—one that relates the motor commands needed to reach the cave with the sensory input expected upon arrival. The process also runs in reverse. If the bat perceives a new settlement and thereby acquires sensorimotor information about reaching it, this egocentric representation can be transformed into an allocentric one. The bat converts its sensorimotor information into a vector originating at its self‐token and ending at a new token standing for the settlement (represented in Fig. 3 by an empty circle), thereby adding this location to its cognitive map.

Borrowing a term from Grush (2000; see also Ismael, 2008), I call an allocentric representation *coordinated* when a self‐token establishes links to and from sensorimotor representations.^4^ Roughly speaking, coordination is the lining up of the allocentric representation with the rest of the cognitive system, such that the representation entails, and is entailed by, sensorimotor information. As it is the relations between self‐tokens and object‐tokens that entail, and are entailed by, egocentrically represented information, coordination necessarily involves a self‐token.^5^

Both Grush's (2000) account of coordination and my own operate at an abstract computational level. In the domain of spatial cognition, a rich literature explores the neurocognitive mechanisms that implement these computations in human and nonhuman brains. While much of the research following the discovery of place cells (O'Keefe & Nadel, 1978) has concentrated on allocentric representation, researchers have consistently recognized the necessity of linking allocentric and egocentric systems. Such translation, I have argued, necessarily requires coordination. Various accounts now illuminate the cognitive and neural mechanisms that underwrite these links between egocentric and allocentric representations.

A classical yet influential account of rodent spatial cognition illustrates how information may be translated between egocentric and allocentric representational formats (Touretzky & Redish, 1996; see also Klatzky, 1998). This model distinguishes five main components. The visual‐perceptual component provides egocentric distance and bearing to perceived objects, drawing on the *what* and *where* streams of early vision. Meanwhile, the head‐direction system tracks the animal's heading in an allocentric format using landmarks and vestibular signals. The path integrator tracks allocentric position^6^ based on information from motor commands’ efference copies, vestibular signals, and optic flow. The final two components synthesize this information. The local‐view component takes inputs from the visual‐perceptual and head‐direction components and constructs a representation that gives distances to objects egocentrically but represents their bearing allocentrically. Finally, the place‐code system merges the local‐view output with path integrator data, yielding a fully allocentric representation.^7^

Such accounts of neural coordination reveal two complications that my abstract framework tends to obscure. First, the boundary between egocentric and allocentric representation proves far from neat. What we find are complex architectures where individual elements implement hybrid representational formats—the local‐view system, for instance, blends egocentric and allocentric features. Second, coordination is established not at a single juncture, but rather emerges in the interplay of various components using a variety of informational streams. For instance, the interaction between the visual‐perceptual and local‐view components coordinates bearing, while the coordination of distance occurs when information from the local‐view and path‐integration components is integrated within the place‐code component. In fact, the distributed nature of coordination runs deeper than the model initially suggests. Consider how the path‐integration and head‐direction components establish allocentric location and bearing, respectively. Both utilize egocentric inputs to update their representations, thereby engaging in their own forms of coordination. What emerges is a highly complex picture where coordination becomes a property of the system's overall functioning rather than any individual step.

However, note that no matter how distributed and piecemeal coordination happens to be, there must be functional integration between the relevant egocentric and allocentric representations for coordination to happen. Here, some recent research on the links between egocentric and allocentric representations in rodents is particularly relevant (LaChance, Todd, & Taube, 2019; Long, Bush, Deng, Burgess, & Zhang, 2025). The neural realizations of both of these formats of spatial representation have received ample attention, and we now know that allocentric representations are realized with the help of place cells in the hippocampus and grid, border, and head‐direction cells in the medial entorhinal cortex. Egocentric representations, in contrast, have been found in the dorsomedial striatum and the parietal, lateral entorhinal, postrhinal, retrosplenial, prefrontal, and sensory cortices. For instance, center‐distance and center‐bearing cells—which represent the distance and bearing to the center of the local environment—have been found in the postrhinal cortex, while egocentric boundary cells—that track the angular offset at which there is an environmental boundary—have been found in the postrhinal cortex and other areas. However, the links between egocentric and allocentric representations have remained elusive. Recently, however, Long et al. (2025) have found egocentric representational cells—both egocentric center‐distance and center‐bearing cells and cells responsive “to a conjunction of allocentric head direction and egocentric bearing” (Long et al., 2025, p. 2)—in the lower levels of rodents’ medial entorhinal cortex, where earlier findings only found allocentric cells. The authors surmise that the medial entorhinal cortex “may be one locus of coordinate transformations between spatial reference frames in the brain” (Long et al., 2025, p. 9). Other researchers have proposed different locations for coordination, such as the postrhinal cortex (LaChance et al., 2019) and the circuits between the retrosplenial and entorhinal cortices (Wijngaarden, Babl, & Ito, 2020).

A coordinated allocentric representation fulfills both conditions for self‐representation: it explicitly represents system properties and directly links them to behavior and sensation. So far, I have only given a sketch of the argument; the rest of the paper fills in the gaps. Over the next few sections, I introduce instances of allocentric representation of temporal, bodily, and cognitive states. Following this, I argue why such representations are genuine self‐representations.

## 2 Allocentrically representing temporal states

Several authors discuss the distinction between egocentric and allocentric representation in temporal cognition (Grush, 2008; Kort, Dickinson, & Clayton, 2005; McCormack, 2015). Many also argue that agents need to—or at least often do—*self*‐represent temporal properties (Campbell, 1999; Goldie, 2012; Lamarque, 2004; Menary, 2008; Peacocke, 2014), making it important to show that allocentric representations can account for these representations of temporal properties. I will proceed in two steps: first, I examine how egocentric representations of temporal properties fail to support certain complex behaviors, and then I turn to allocentric representations and their links to self‐representation.

When food becomes scarce, slime mold amoebae merge into plasmodia that forage for nutrients. Saigusa, Tero, Nakagaki, and Kuramoto (2008) exposed these plasmodia to pulses of dry conditions, knowing they slow down in dry environments. After three exposures, slime molds begin to periodically slow down even without the dry condition stimulus, indicating they have begun to *anticipate* them.

To behave anticipatorily, slime molds track time using regular internal oscillations (Saigusa et al., 2008). These oscillations encode a〈*timespan*〉 variable tracking temporal distance to the next dry condition. Using such *interval timing* (Buhusi & Meck, 2005), a slime mold can slow its movement when 〈*timespan*〉 approaches zero.

Significant parallels between path integration and interval timing suggest that both use egocentric representations. Where 〈*timespan*〉 specifies the temporal distance *from the present*, 〈*distance, bearing*〉 specifies the spatial distance and bearing *from the current location (and orientation)*. In both cases, the representations concern (but are not about) relations *from the system's actual state*. Hence, egocentric temporal and spatial representations require updating with each state change. Path integration updates spatial representation, while internal oscillations decrement 〈*timespan*〉 to track time.

Egocentric temporal representations are restricted to representing properties with a single token concerning a relationship to the present. They “are not carrying contents to the effect that this or that thing is happening at this or that time” (Grush, 2008, p. 156) and cannot explicitly represent relations between moments in time. Consequently, they cannot represent an event as happening before or after another (except tacitly, when one of the events is in the present). McCormack (2015) calls this a conception of *time as duration*, as the explicitly represented content is a duration from the present to some other moment.

Grush and McCormack contrast this form of temporal cognition with “a more sophisticated kind” (Grush, 2008, p. 156) that explicitly represents relations between events. A study by Arcediano, Escobar, and Miller (2003) illustrates this. They first exposed rats to two neutral stimuli (two sounds in this case) following one another with a 5‐s gap (*S2* → *S1*). In a second phase, the same rats were exposed to an aversive stimulus *US* (a footshock) paired with *S1* (*US* → *S1*). In the test phase, the rats were exposed to *S2*. The rats displayed decreased use of a lick bottle following this exposure, demonstrating a fear response.

What is notable about the rats’ response is that they seemed to anticipate the footshock on exposure to *S2* even though they had not been conditioned on the relevant sequence (*S2* → *US*). This, the study's authors assert, shows that rats can integrate the two learned temporal sequences to arrive at a sequence *S2* → *US* → *S1*, leading the rats to expect *US* on being exposed to *S2*. To do so, they think, requires a temporal map, a kind of cognitive map already proposed by Tolman (1948).

Why might an allocentric temporal representation be needed? Egocentric representations of temporal properties could be used to measure the duration of the events taking place in the first two phases of the experiment. In the first phase, a representation *R1_ego_* could be instantiated to measure the duration between *S2* and *S1*. In the second phase, another representation *R2_ego_* would measure the duration between *US* and *S1*. For both representations, the fact that they are relations *between* certain events is only tacit in their use (i.e., tacit in their activation at *S2* and *US*, respectively, and their subsequent employment in behavior that is anticipatory of *S1*). That is why, even though *R1_ego_* and *R2_ego_* carry explicit content about a relation to *S1*, the rats cannot, say, subtract the value in *R2_ego_* from *R1_ego_* to arrive at the temporal distance between *S2* and *US*. Or, more precisely, while they *could* subtract one value from the other, they would not thereby have any indication of how to use the resulting representation; they would not, unlike in the case of *R1_ego_* and *R2_ego_* have formed a nonrepresentational association between a certain event (here, *S2*) and activation of the token.^8^

To integrate the two temporal sequences, rats must represent them allocentrically. When their representations *R1_allo_* (about *S2* → *S1*) and *R2_allo_* (about *US* → *S1*) explicitly represent relations between events, they may integrate representations that share an element (Molet, Miguez, Cham, & Miller, 2012,). In the experiment, rats first learn that *S2* precedes *S1*. Then, in the second phase, they learn that *US* precedes *S1*. Because *S2* does not occur between *US* and *S1*, they seem to infer that *S2* occurs before *US*, arriving at *S2* → *US* → *S1*.

In an allocentric representation, time may be said to function as a framework (McCormack, 2015) that organizes the temporal relations between events. These relations do not make tacit reference to the system's actual state, the present. This is evidenced by the fact that the rats’ representation of *S2* → *US* → *S1* may remain accurate as time passes, even if it is not updated. In other words, temporal relations are represented in a way that is in principle independent of the system's actual state.

Since allocentric temporal representations do not necessarily concern the system's state, coordination becomes an issue. A representation of *S2* → *US* → *S1* does not indicate how these events relate to the present, that is, the rats’ actual state. Just as creatures may be ignorant of where they are, they may be ignorant of when they are. It is only when *S2* occurs that a rat can learn that it is temporally colocated with *S2*, coordinate the allocentric representation with sensorimotor information, and anticipate *US*.

Research on the neural mechanisms of temporal cognition lags considerably behind research in spatial cognition. Even in spatial cognition, the connections between egocentric and allocentric representational formats remain poorly understood—and these links are still more obscure in temporal cognition. The very notion of allocentric temporal representations remains contested, particularly for nonhuman animals (Suddendorf & Corballis, 2007). In humans, allocentric temporal representations are studied as *episodic memory*, which is thought to involve the hippocampus as does allocentric spatial cognition. However, how such representations connect to egocentric temporal representations—such as those modeled by pacemaker‐accumulator accounts (see Gür, Duyan, & Balcı, 2018)—remains unclear.

If my account proves correct, we should find analogues of the path integrator (or head‐direction) component in temporal cognition. These would locate animals within allocentric time so that egocentrically represented events can be converted into an allocentric format and vice versa. Coordination is, in some ways, easier to achieve in the temporal domain since self‐initiated change of temporal properties is impossible. A pacemaker‐accumulator system, supplemented by periodic recalibration through external cues such as solar and celestial signals, can effectively maintain one's position in allocentric time. Further empirical research must illuminate how these processes operate within human and nonhuman cognitive architectures.

Coordinated allocentric representation of temporal properties links to self‐representation. A creature with such a representation explicitly differentiates between its actual temporal properties and those of past and future events—including, potentially, its own past and future states. Such representation is *about* the system instantiating it. Through coordination, the system uses a certain token in a special way, namely, as a self‐token that specifies the system's actual temporal state. The system is disposed to infer sensorimotor information from relations between the self‐token and other tokens and to update the representation based on sensorimotor information. This suggests that creatures with allocentric temporal representations are genuine self‐representers.

## 3 Allocentrically representing bodily states

Many animals represent their own bodies. These representations develop early in ontogenesis (Meltzoff, Saby, & Marshall, 2019) and prepare the ground for representing the body‐external environment (Stoytchev, 2009). Some of these representations are allocentric and suggest a link to minimal self‐representation. Unlike previous sections, I will turn to allocentric representations without contrasting them with their egocentric counterparts.

Infants begin successfully reaching for objects at 3−4 months. Initially, their reaches are jerky and inaccurate, not following straight lines (Hofsten, 1982; Thelen, Corbetta, & Spencer, 1996). By 5 months, infants anticipatorily adjust their hands to object shapes (Hofsten & Fazel‐Zandy, 1984; Witherington, 2005), and by 7 months, they can reach efficiently (Thelen et al., 1996) and drink from open cups (Hofsten & Fazel‐Zandy, 1984). At 2 years, their motion smoothness approaches adult levels (Berthier & Keen, 2006).

Researchers initially attributed early jerky movements to vision‐guided reaching (see Corbetta, Wiener, Thurman, & McMahon, 2018), theorizing that infants paused to check and correct their motions by gazing back and forth between hand and target. They thought smooth movements emerged only later, with the development of a sense of the body. This view has been largely abandoned. Hofsten and Lindhagen (1979) found that infants fixate on the target object rather than alternating their gaze. More decisively, Clifton, Muir, Ashmead, and Clarkson (1993) demonstrated successful reaching even when infants cannot see their hands and arms, indicating they must already possess an embodied sense of hand location (Corbetta, Thurman, Wiener, Guan, & Williams, 2014).

Converging evidence from developmental psychology and robotics (Baranes & Oudeyer, 2013; Desmurget & Grafton, 2000; Schillaci, Hafner, & Lara, 2016) indicates that infants’ developmental trajectories and emergent abilities can be explained by internal bodily models: *forward models* and *inverse models*. Forward models (Grush, 2004; Wolpert & Ghahramani, 2000) allow systems to compute the bodily (and sensory) state to which a motor command will likely give rise. As forward models represent the bodily dynamics internally, they can be used to estimate states even when actual sensory input is noisy. Moreover, because forward models are realized in the brain, their predictions are available long before proprioceptive signals have finished their trip back from the sensory surfaces. With a forward model, an infant could, say, predict that the motor commands it just issued will cause it to fail to reach a toy in front of it. Importantly, the infant could do so in the absence of—and long before—visual sensory input that confirms this. Inverse models—being the inverse of forward models—allow computing the motor commands required to reach a target state and thus facilitate goal‐directed behavior. This could free up the infant from needing to constantly visually line up the hand with the target in order to compute the motor commands necessary to reach the toy.

Forward and inverse models need information about the system's current bodily state. For instance, depending on whether her arm is flat against the side or stretched out in front of her, an infant's inverse model should issue different predictions about the motor commands necessary to reach a toy. The forward model, too, will predict different bodily states depending on the infant's current posture. Hence, the forward model relates possible bodily states in terms that may be inferred from motor commands, whereas the inverse model relates possible bodily states in terms that allow inferring the motor commands required to transition between them.^9^

Internal body models are allocentric representations. Not only do internal body models explicitly differentiate between various possible states, but the relations these bodily states are represented to bear on one another are, in principle, independent of the system's actual posture. When such a representation contains a token giving the system's actual state, it is thereby *about* the system's actual state.

To employ such a model effectively, a system must designate one of its body state tokens in a special way—as representing its own actual state. As Coslett, Buxbaum, and Schwoebel (2008) put it, “you are here” information is required “to accurately reach toward an object” (p. 117). In other words, an internal body model needs to be coordinated for the system to use it. This coordination operates bidirectionally between egocentric and allocentric representations. In one direction, efference copies of motor commands as well as sensory feedback—both in egocentric formats—drive updates to the allocentric body model in the cerebellum so that it represents the system's current state (Grush, 2004). In the opposite direction, the system infers (egocentric) motor commands from the (allocentrically) represented relations between the self‐token and tokens representing merely possible bodily states.

Coordinated allocentric representation of bodily states suggests a form of self‐representation. To allocentrically represent bodily properties means—in the case of the inverse model—to represent oneself as exemplifying a certain posture that is explicitly differentiated from other postures the system could exemplify if it were to issue certain motor commands. It is *about* the system's actual state (and does not merely concern it). Reminiscent of certain allocentric temporal representations, the *other* from which the system is explicitly differentiated here is the system's own merely possible state.^10^

## 4 Allocentrically representing cognitive states

We routinely represent ourselves to have beliefs, desires, and other mental states, and some authors consider this ability necessary for self‐representation (see Musholt, 2013). Here, I show that there are no principled difficulties in extending my account to such properties and demonstrate that such representations exist in certain nonhuman animals.

Recent experiments highlight sophisticated forms of social cognition in corvids, particularly scrub‐jays (for instance, Bugnyar, Reber, & Buckner, 2016; Kort et al., 2005). Many of these studies make clever use of scrub‐jays’ predilection for caching food and pilfering conspecifics’ caches. One such experiment, by Clayton, Dally, and Emery (2007); see also Nathan J. Emery & Clayton, 2008), studied how a conspecific observer's presence during cache recovery affects the cacher's behavior. During a first caching event, an observer (A) watches through a transparent partition as the cacher hides food in tray A (tray B being visible but inaccessible). At a second event, a different observer (B) watches the cacher hide food in tray B (tray A now being inaccessible).

At recovery, cacher behavior depends on which observer is present. With observer A present, cachers frequently move the food previously hidden under tray A to a new cache, often moving it back and forth multiple times, while leaving tray B's contents untouched. Thereby, the cacher seems to attempt to protect the food observer A saw being cached while not revealing the food the observer does not know about. With observer B present, the behavior is analogous but targets the other tray.^11^

The study's authors believe the experiment to show that corvids represent conspecifics’ mental states, though others (Penn, Holyoak, & Povinelli, 2008) consider such conclusions premature. These detractors argue the experiment fails to demonstrate that corvids exhibit “a sensitivity to what others have and have not seen” (Clayton et al., 2007, p. 519) or engage in “knowledge attribution” (ibid.): The caching bird might simply represent which observer was present at which caching event and recache food accordingly. Scrub‐jay behavior could then be explained by positing that corvids represent spatial and temporal properties, and not conspecifics’ cognitive states.

However, only birds that have pilfered others’ caches engage in cache protection, which suggests an alternative explanation (N. J. Emery & Clayton, 2001). Corvids appear to infer others’ behavioral patterns from their own, and this requires that they distinguish between their own informational states and others’ informational states. Only then can the cacher differentiate between where it thinks the cache is located and where the conspecific thinks it is located. The cacher must recognize these informational states as being of the same kind, allowing it to understand that these states guide behavior similarly. Thus, scrub‐jays must represent that conspecifics can be in informational states of the same *kind* but differing in *value* from their own.

Butterfill and Apperly (2013) propose that scrub‐jays distinguish between their own and others’ spatial representations without representing mental states. Instead, they represent *registrations*, where “an individual registers an object at a location if and only if she most recently encountered it at that location” (Butterfill & Apperly, 2013, p. 617). Like beliefs, registrations guide behavior and can be incorrect. Through registrations, a scrub‐jay can predict others’ behavior across many (though limited^12^) situations. Seeing a conspecific register food at a cache location, a cacher can anticipate attempted pilfering.

Scrub‐jays represent registrations as 〈*individual, location, object*〉 tuples. At cache recovery, they select registrations matching the present conspecific and location. The cacher then acts to ensure that the *location* and *object* elements of these registrations do not match its own representation of cache location. For example, if a scrub‐jay represents food to be under tray A while a conspecific has registered it there, it will move the food to a new location.

It is still murky at this stage how a scrub‐jay compares a conspecific's registrations with its own representation of cache locations. After all, and as I have mentioned above, for the cacher to infer the observer's behavior from its own, the informational states involved in the two cases need to be comparable. However, registrations are not like the scrub‐jay's own representations of spatial properties and, therefore, cannot be directly compared to these. To enable comparison, the scrub‐jay needs to translate information stored in their representation of spatial properties into 〈*individual, location, object*〉 tuples where the *individual* is it itself. These registrations can then be compared to those of conspecifics. How this is done does not matter for our purposes; what matters is that translation is required for the scrub‐jay to render others’ registrations different from its own representation of spatial properties.

The mechanisms coordinating representations of cognitive states remain even more speculative than those governing spatial or temporal properties. Intriguingly, the hippocampus and entorhinal cortex—both central to allocentric spatial representation—also activate during cognition of diverse properties: values, sound frequencies, social hierarchies, reward probabilities, and more (Whittington, McCaffary, Bakermans, & Behrens, 2022). This suggests that the neural mechanisms enabling allocentric spatial representation may also map relations between other property types—possibly including cognitive properties. While these possibilities prove tantalizing, crucial questions persist, particularly regarding the mechanisms that convert allocentrically represented information into egocentric formats and vice versa. The present analysis implies we should expect circuits mediating between egocentric and allocentric representational formats for representations of cognitive properties, analogous to how the medial entorhinal cortex (and/or postrhenial and retrosplenial cortices) functions in spatial cognition.

This representation matches the pattern seen in earlier coordinated allocentric representations. Various tokens represent objects (individuals) and their properties (registrations) in a way that is in principle independent of the system's own state. This independence means that for the system to employ the representation, it must use one specific token as specifying its own state. Once a self‐token is used to coordinate the representation of registrations with sensorimotor information, the scrub‐jay can update the representation based on sensory information regarding its own and others’ registrations, and it can use allocentrically represented information to infer what behavior is appropriate in some specific observer's presence.

As with other allocentric representations, the representation of properties with coordinated allocentric representations has intriguing links to self‐representation. A scrub‐jay explicitly differentiates between where it and where others have encountered objects. The representational token that specifies its own registrations is *about* itself. Relations between its own registration and those of others imply sensorimotor information, as evidenced by scrub‐jays’ caching behavior. Such allocentric representation, hence, seems to imply a form of self‐representation—scrub‐jays seem to represent themselves (and others) as exemplifying cognitive states.

## 5 Minimal self‐representation

In previous sections, I argued that coordinated allocentric representations are used by a range of creatures to represent various kinds of property. I now show why *any* coordinated allocentric representation is a minimal self‐representation: They all fulfill the two conditions on self‐representation. They explicitly attribute properties to the representer, and the representer knows the representation is about itself. In other words, such representations are, first, *about* the representer and, second, have genuine *de se* content.

To be about the representer, an allocentric representation must explicitly represent the system's own state. It does so when it contains a (mere) self‐token: an object‐token that stands for the system. With such a token, the fruit bat may represent its location and the scrub‐jay its registrations. In such representations, the self figures as an *articulated* constituent used to explicitly differentiate between self and other. This other from which the self is differentiated need not be another object: as internal body models have shown, the other may refer to the system's own merely possible states.

A (mere) self‐token is not sufficient for a representation to have *de se* content. Self‐representation additionally requires that the representer knows that it* is the object represented by the self‐token. A fruit bat's cognitive map may include its location, but as long as the bat fails to know that this token represents its* location, the bat does not self‐represent. Similarly, for a rat to self‐represent its temporal properties, it must know which of the represented moments in time is its* temporal state (i.e., the present).

A representer knows some token is about itself when it knows how to use the representation to guide its behavior and how to update the representation based on sensations. Only when the bat knows where it* is can it use its representation for locomotion and update the representation based on perceptual input. Only when the rat knows when it* is can it use its representation to anticipate events and temporally represent new events based on perceptual input.

According to my proposal, this form of knowledge‐how comes about when an allocentric representation is *coordinated*. Coordination establishes systematic nonrepresentational—or architectural (Ismael, 2008)—relations between sensorimotor and allocentric representation, enabling the representer to infer sensorimotor information (which are in an egocentric format) from allocentric representation and vice versa. It is through coordination that a representer learns what token represents its* properties, and it is hence through coordination that the representer instantiates a self‐representation.

Coordination revolves around a self*‐token. This is a special representational token through which a system represents itself in a way that directly links to behavior and sensation. Unlike (mere) self‐tokens, a self*‐token is individuated not by what it represents but by how the cognitive system *employs* it. Consider spreadsheet software: selecting a cell highlights it, indicating which piece of data your next command will affect. Like this highlighting, a self*‐token does not *add* information but indicates which token is linked to the system in a special way. A self*‐token denotes that token in a self‐representation that is used by the system in the special way that manifests the system's know‐how that this token stands for itself.

More specifically, a system knows the self*‐token is about its* state when it employs the token in inferences to and from sensorimotor information. For an allocentric representation to imply sensorimotor information, one of its tokens must be *used as* denoting the system's state. If a fruit bat is to infer the motor commands needed to get home, it must instantiate, in addition to the object token about the cave, a self*‐token that indicates its* own position. A rat may only use its allocentric representation to predict future events when it uses one of the represented moments in time as its* temporal state. And it is only when an infant uses one of the many bodily states represented by an internal model as specifying her* actual bodily state that she may employ an inverse model to infer the motor commands required to reach some goal state.

Since a creature cannot use its allocentric representation without a self*‐token, reference to the self*‐token is necessary to explain how the representation issues in behavior. This parallels the observation by Castañeda (1966) and Perry (1979) that certain indexicals are essential to explain actions (see also David Lewis, 1979; Mach, 1890).^13^ Consider the seminal example by Perry, in which he imagines following a trail of sugar around the supermarket until he realizes that he* is the one with the torn sack. It is impossible, Perry says, to explain why he adjusted the torn sack in his cart without referring to him realizing that he* is the one with the torn sack. Replacing “he*” with descriptions like “the only bearded man in the store” fails to explain the behavior if we do not attribute to Perry the knowledge that he* is that man. Similarly, explaining a scrub‐jay's recaching requires reference to a self*‐token. No amount of allocentric information (even including mere self‐tokens) allows the bird to infer sensorimotor information without a self*‐token. Only when the representation is coordinated, and a token is specified as self*‐token, does the cognitive system know the origin of the allocentrically represented relation that implies sensorimotor information.

Links in the opposite direction—from egocentric to allocentric representation—are also crucial for self‐representation. They allow the system to update its allocentric representation with information from the senses. A rat with sensorimotor information about some future event can only encode that information in its allocentric representation if it knows which representational token is about its* temporal state. An allocentric token's value can only be inferred based on sensorimotor information *and* the self*‐token. Only then can the rat establish a correspondence between the sensorimotor information and a *relation* from its* temporal state (i.e., the self*‐token) to the event. The same applies to the other creatures we have looked at: on having gained sensorimotor information about the motor commands required to, say, lift her arm, an infant may want to use this information to enrich her internal model. Since the sensorimotor information corresponds to a relation between her* actual state and some goal state, a self*‐token is necessary to update the allocentric representation.

I have argued that allocentric representations explicitly differentiate between properties attributed to the self and properties attributed to others, while coordination links such representations to sensorimotor information. These links entail, first, that a creature with a coordinated allocentric representation is disposed to take the represented information as directly relevant to its behavior. Second, the links entail that the creature is disposed to update the representation based on information supplied by the senses. Such a creature knows it represents itself and hence instantiates a self‐representation.

*Any* creature representing *any* property with a coordinated allocentric representation is a self‐representer. This means that the scrub‐jays, fruit bats, young infants, and rats I discussed throughout the paper are all self‐representers.

## 6 How to find out who you are

A crucial question remains: how does a system determine which token to use as a self*‐token to coordinate allocentric representation with sensorimotor information? In this section, I show how a creature can gain information about itself in two ways. First, there is the subjective route, which involves making explicit the unarticulated constituent of a sensorimotor representation. Second, there is an objective route, where the representer identifies itself with an allocentrically represented object. I show that self‐attributions of the first kind are immune to certain errors through misidentification, which many philosophers consider essential for self‐representation (Evans, 1982; Musholt, 2013; Shoemaker, 1968).

The most important way a system can learn about its* properties is via the subjective route—by establishing correspondences between sensorimotor information and *relations* in the allocentric representation. This establishes coordination between these representational formats so that information may be converted from one to the other.

**Fig. 4:**
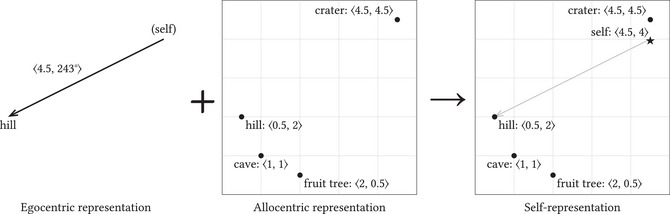
Inferring a self*‐token from sensorimotor information.

Consider spatial cognition as an example of such coordination (Fig. 4). A creature representing distance and bearing to an object can use its allocentric spatial representation to determine its own location—provided it allocentrically represents its head‐direction. Alternatively, if the system lacks an allocentric representation of head‐direction, the creature can either triangulate using multiple landmarks or encounter a known location so that it can represent itself as being at that location. Animals intelligently combine information from path integration, head‐direction tracking, landmarks, and so forth, weighing each source according to its estimated reliability (Charles R. Gallistel, 2008).

My account implies that similar mechanisms exist for the representation of the various other properties that creatures self‐represent. The basic idea is always the same: Find relations in the allocentric representation that imply the egocentrically represented information that you have access to. Sometimes multiple states in the allocentric representation may do so, in which case additional information is required. Such additional information may be in an allocentric format (such as information about head‐direction provided by head‐direction cells) or an egocentric representation (such as when using multiple egocentrically represented 〈*distance, bearing*〉 tuples in triangulation). For instance, an infant may possess sensorimotor information linking various motor commands to bodily movements. She can then use this information to search her internal body model for a token whose relations to various object‐tokens imply the sensorimotor information she possesses. She can then use this token as self*‐token. Just as a system may infer sensorimotor information from relations between a self*‐token and object‐tokens, it may do the reverse and infer a self*‐token from sensorimotor relations.

If my account of self‐representation proves accurate, we should expect coordination mechanisms for all self‐represented properties. Each such property requires a mechanism that translates between allocentric and egocentric representations. This yields testable predictions. First, functionally differentiated circuits should exist for egocentric and allocentric representations, activated differentially depending on task demands. Circuits realizing only egocentric representation should be capable of underwriting limited behavioral capacities independently of the activation of allocentric representations. Tasks requiring the allocentric representation of certain properties without requiring the self‐representation of these properties should activate only allocentric circuits (more on this toward the end of this section). Finally, we should expect to discover neural circuits realizing both representational formats that are involved in translation between them. Such circuits should activate whenever coordination occurs and, consequently, whenever self‐representation emerges.

Such coordination is possible because sensorimotor information concerns the self. When matching sensorimotor information to a relation in the allocentric representation, the object‐token at the relation's origin articulates the sensorimotor representation's unarticulated constituent. Since the unarticulated constituent refers to the self, its articulation as an object‐token also refers to the self, allowing the system to designate that token as a self*‐token without possibility of error.

Since such self*‐tokens cannot attribute properties to the wrong object, they are immune to errors through misidentification (IEM) in the same way as certain uses of the indexical “I” (Evans, 1982; Perry, 2010; Shoemaker, 1968; Wittgenstein, 2007). These uses of “I”—what Wittgenstein (2007) called uses of “I” as subject—are such that it is impossible to ascribe a property to the wrong person. When thinking “I see a tree” (or “I have a headache”), it makes no sense to wonder, “Someone is seeing a tree, but is it *I*” (or “Someone is having a headache, but is it *I*”). I might be mistaken about the content of the visual experience, but the fact that *I* am having the experience is beyond doubt. The matter is no different with self*‐tokens inferred from sensorimotor information: while they may misrepresent a system's properties, they necessarily refer to the system itself. Coordinated allocentric representation “allows for the possibility that it misrepresents the property that is being ascribed, while it cannot misrepresent the subject purportedly possessing that property” (Musholt, 2013, sec 2.3). A bat might infer its self*‐token from inaccurate sensorimotor information, leading to a misrepresentation of its properties in the allocentric representation. However, since sensorimotor information is necessarily self‐concerned, the self*‐token cannot but refer to the system itself.

My explanation of why certain self‐ascriptions are IEM is similar, in certain ways, to the account advanced by Recanati (2009, 2012, 2024). Like me, Recanati argues that the status of certain self‐ascriptions as IEM derives from the content of the grounds for those self‐ascriptions. It is because the contents are of a certain kind that a system need not identify with some object to self‐ascribe a property, hence making mistakes through misidentification impossible. However, Recanati further argues—and here we part ways—that self‐ascriptions are IEM when grounded in experiences with a certain kind of *mode* (see Searle, 1983). The mode of an experience phenomenally distinguishes experiences of different kinds without being part of the experience's content. For instance, proprioception's functional role is to provide information about one's body, and because of this functional role, such experiences feel a certain way. Importantly, such experiences are selfless, so that when I experience my legs being crossed, I just experience *crossed legs*. While the self does not figure in the content of the experience, an agent can identify the kind of experience she is having by its mode, and given that experiences of this mode necessarily provide information about the self, “[t]he person in question is, as it were, pre‐identified, being determined by the mode of the experiential state” (Recanati, 2024, p. 9). Hence, I cannot misattribute the property when I move from a proprioceptive experience of crossed legs to attributing to myself the property of having crossed legs.

This approach faces a difficulty: why can *external mode* experiences—such as perception—also ground IEM self‐ascriptions even though their role is not to provide information about the self? Recanati argues this is because these experiences are also “bound to be about the subject of experience” (2024, p. 10). When seeing the Eiffel Tower, it is necessarily present *in the subject's environment*. Thus, my belief that *I am standing in front of the Eiffel Tower* is IEM. However, note that the mode no longer does any work: experiences of the internal and external mode can ground IEM self‐ascriptions.

In my view, self‐ascriptions are IEM when grounded in egocentric representations and, since experiences of the internal *and* external modes can be egocentrically represented, both may provide the relevant grounds. The crucial factor is not that egocentric representations are selfless—when I look in a mirror, I see *myself*, yet this can ground the IEM self‐ascription *I stand in front of a mirror*. Rather, IEM stems from the fact that these representations are *self‐concerning* (viz. Ismael, 2012); whenever a self‐ascription articulates an egocentric representation's unarticulated constituent, it is IEM.^14^

The second—objective—way of finding out who you are is not immune to errors through misidentification. Whether an allocentrically represented property is IEM depends on the grounds for the self‐ascription (Evans, 1982). If, after an accident, I (visually) perceive a broken arm and form the belief that my arm is broken, this belief is not IEM. The arm I am seeing may not be my arm and, hence, the person with the broken arm may fail to be me. Here, my judgment that I have a broken arm is ultimately based on the articulated (explicit) object constituent of a sensorimotor representation. My seeing of the broken arm might instantiate sensorimotor information to the effect that if I moved my head this or that way, I would see it from this or that angle. I could then use this information to add an object‐token to my allocentric representation and thereby represent a person with a broken arm. I might then, for some reason or other, judge that I am identical with this person. I would now instantiate a self*‐token that attributes to me the property of having a broken arm. As this judgment of identity is fallible, the resulting self‐ascription is not IEM. We can compare this situation to one in which I use proprioceptive information to determine that my arm is broken. This judgment is IEM since it is based on grounds of a different kind, namely, a sensorimotor representation's unarticulated constituent.

Note that at least *some* self*‐token values must derive from the articulation of an egocentric representation's unarticulated constituent. A judgment of identity between a self*‐token and some object‐token obviously requires a self*‐token. If this self*‐token's value is itself derived from a judgment of identity with another self*‐token, then that requires yet another self*‐token. This well‐known infinite regress (Evans, 1982; Peacocke, 2014; Shoemaker, 1968) can only be stopped by a self*‐token deriving from a sensorimotor representation's unarticulated constituent.

I do not mean to suggest that *every* use of an allocentric representation requires coordination with egocentric ones. Imagine that fruit bats could communicate food locations to their conspecifics so that a bat could inform another that fruit tree A lies closer to the cave than fruit tree B. To do so, the bat does not need to know its location—it is enough that it knows the spatial relation between the cave and the fruit trees. Yet while such uncoordinated allocentric representations can contribute importantly to behavior, they are never sufficient. The bat cannot infer the relevant sensorimotor contingencies from the uncoordinated allocentric representation alone but needs additional sensorimotor information about the communicative situation. For instance, if it knows that it can expect to be treated favorably in the future if it helps the conspecific, it could then use the allocentric representation to figure out how to help.^15^

To conclude, there are two radically distinct ways of gaining knowledge about oneself. The first of these, the subjective route, is IEM as it involves making explicit a sensorimotor representation's unarticulated constituent. The second, objective route involves identifications with object‐tokens and leads to self‐ascriptions that are not IEM.

## 7 Substantive self‐representation

Minimal self‐representations arise with relative ease because they impose no constraints on which properties must be represented. They require only that some property—any property—be represented through a coordinated allocentric representation. We might worry that this permissive account says too little about the sophisticated self‐representations we find, for instance, in adult human beings. In this section, I suggest that substantive forms of self‐representation develop from minimal ones. The developmental trajectory of self‐representation in children offers a window into this process, showing how my proposal can guide empirical research on the emergence of self‐representation.

First, it will prove useful to contrast my proposal with some of the views espoused in the philosophical literature, where authors have argued that the representation of this or that kind of property is essential for self‐representation. Musholt believes that self‐representation requires “acquisition and application of the first person concept” (Musholt, 2013, sec. 4), which depends on representing one's own and others’ *mental states* (Musholt, 2012). Grush (2000), following Strawson (2011), writes that “the subject/object distinction is the result of a cognizer's representation of space” (p. 62). Peacocke (2014) and Campbell (1999) argue that temporal properties are also necessary. This emphasis on temporal properties is also one of the few points of agreement in the literature on narrative selves (Goldie, 2012; Lamarque, 2004; Menary, 2008). Another group focuses on bodily representations (Hohwy & Michael, 2017; Metzinger, 2003) with Hohwy explaining “self‐representation in terms of inferred hidden causes” (p. 374) that “stem from the organism itself” (p. 375).

My account suggests that these disagreements are not about what constitutes genuine self‐representation, since any coordinated allocentric representation qualifies as such. The dispute may then stem from one of two sources. First, philosophers may disagree about which properties a creature must represent to count as representing a self *qua* self. If we believe that genuine selfhood requires possessing mental properties, then a system fails to represent a self *as a self* when it does not represent these properties. The dispute is then about the metaphysics of selfhood rather than self‐representation as such. Alternatively, these differences might only *appear* to conflict and simply describe distinct—but complementary—kinds of self‐representation. The differences between accounts then mostly boil down to variations in self‐representations’ richness.

This second interpretation proves particularly significant as it illuminates my proposal's potential to guide the empirical study of self‐representation. The account's capacity to provide a principled approach to representational richness—using operationalizable concepts—constitutes one of its primary theoretical advantages. According to this view, different kinds of creatures can deploy coordinated allocentric representations to represent different kinds of property, and individual agents may employ self‐representations of varying sophistication across their lifetimes. This opens compelling research avenues: we can now compare self‐representations across diverse systems—from humans to nonhuman animals to artificial agents—and illuminate the developmental processes that shape self‐representation at both ontogenetic and evolutionary timescales.

In the remainder of this section, I want to show how this framework might illuminate children's development of self‐representation. The classical starting point here is William James’ differentiation between the “I” and the “me” (1992), which marks a distinction between implicit self‐representation and the self‐concept (e.g. Carmody & Lewis, 2012; Southgate, 2024; Woźniak, 2024).^16^ The question about the links between implicit self‐representation and explicit self‐concept “remains a difficult – and largely uncharted – domain” (Southgate, 2024, p. 111). We have little understanding of which “cognitive and neural mechanisms might allow infants to exploit [implicit self‐representation] for developing self‐awareness” (Southgate, 2024, p. 111). Since the dichotomy between “I” and “me” roughly aligns with my distinction between self‐concerning and (explicit) self‐representation, I believe my account can inform research here.

Self‐representation is complex, and one way researchers grapple with this complexity is by refining the basic distinction between “I” and “me.” James himself subdivided the “me” into material, social, and spiritual aspects—a strategy that contemporary theorists have pushed considerably further. Neisser (1991) distinguishes ecological and interpersonal implicit self‐representations from conceptual self‐representation, while Schlicht et al. (2009) proposes an alternative tripartite model. Others have multiplied categories still more: Rochat identifies three (Rochat, 2010, 2013) or five (Rochat, 2003) varieties, Woźniak (2024) roughly four, and Riva (2018) six distinct self‐representations. Though these distinctions often capture genuine insights, they reflect researchers’ particular theoretical commitments rather than principled criteria—a proliferation that complicates meaningful comparison. I believe my account offers a more systematic way to distinguish between kinds of self‐representation.

Moreover, the apparent diversity nonetheless rests on a broad consensus around James’ distinction between implicit self‐representation and the self‐concept. The complications involve subdividing either explicit or implicit forms rather than challenging this boundary itself. The prevailing accounts hold that implicit self‐representation develops first, subsequently giving rise—around 18 months, coinciding with success at mirror self‐recognition—to the self‐concept (Southgate, 2024). This timeline finds support in the coemergence of related phenomena: self‐other comparison (Kampis, Grosse, Charlotte, & Southgate, 2022), perspective conflict detection (Yeung, Askitis, Manea, & Southgate, 2022), empathy (Bischof‐Köhler, 2012), personal pronoun use (Michael Lewis & Ramsay, 2004), and increased connectivity in brain regions linked to self‐referential processing (Bulgarelli et al., 2019).

Before criticizing the common assumption that a unitary self‐concept emerges at a very specific moment in development, I want to emphasize that there is much that is right about the received view. First, there genuinely exists a differentiation between implicit and explicit self‐representation—after all, this has been one of the main arguments in the present paper. Second, as many in the literature argue, particularly Neisser (1988, 1991) and Rochat (2001, 2003, 2010, 2013), this distinction marks the difference between representing a world of mere affordances and taking oneself as an object differentiated from others.

While I agree that self‐representation involves a capacity to take oneself as an object, this capacity does not emerge all at once. There is no singular moment when *the* self‐concept arises. Rochat illustrates the received view through an onion analogy: different kinds of self‐representation—particularly implicit and explicit forms—constitute layers that build upon one another (Rochat, 2003, 2013). This suggests a neat progression from one kind of self‐representation to the next, with earlier forms providing foundations for subsequent levels. I argue instead that explicit self‐representation arises when *any* property becomes represented through coordinated allocentric representation. This means an infant may self‐represent certain properties before other properties. Rather than discrete layers, we find a complex web of strands, each potentially shifting from egocentric to allocentric representation at different moments. Instead of the neat layers of an onion, imagine a garden in spring: different plants sprout and flourish at their own pace, entering into complex relationships with neighboring growth at various stages of development. Some flowers bloom early, while others remain dormant; some plants provide support for climbers that emerge weeks later. It is the garden's state as a whole—the particular constellation of egocentric and allocentric representations at a moment—that determines the system's cognitive and behavioral capacities. Self‐representation thus emerges piecemeal and gradually—not through a single decisive transition from implicit to explicit forms.

The view that explicit self‐representation emerges gradually gains support from the fact that different properties seem to be represented by coordinated allocentric representation at markedly different ages. Allocentric body representations develop very early, as do allocentric spatial representations (Lew, Bremner, & Lefkovitch, 2000). Similarly, infants can distinguish between their own and others’ attentional states well before 18 months—a capacity requiring coordinated allocentric representation (Hauser, 2025). Other forms of self‐representation arise much later: for instance, even 18‐month‐olds cannot take themselves as the object of (potentially false) beliefs. This timeline challenges the assumption that self‐concept emerges at a discrete moment in time.

This questionable assumption has two important consequences. First, developmental psychologists may not be studying what they think they are studying. Having misspecified the research *explanandum*, they use mirror self‐recognition as a test for the self‐concept when it at most tests for a specific kind of self‐representation. Zahavi and Roepstorff (2011) already highlighted this conflation, critiquing developmental psychology's move “from the experiment as a model *of* a particular notion of the self to the experiment as a model *for* Self” (Zahavi & Roepstorff, 2011, p. 142, emphasis in the original). The proposed account addresses this confusion by offering a philosophically sophisticated and empirically operationalizable notion of self‐representation—something currently absent from both developmental psychology and philosophy.

Second, the Jamesian division between “I” and “me” forestalls promising accounts of how self‐representation develops. The strict dichotomy makes it difficult to explain how explicit self‐representation emerges from implicit roots. Researchers often resort to vague language that betrays this conceptual difficulty. Rochat, for instance, speaks of the 9‐month miracle marking the “beginning of the” self‐concept (Rochat, 2013, p. 386), yet claims that “[s]elf‐objectification as a new level of self‐conceptualizing appears to emerge unambiguously from approximately 14 to 18 months” (Rochat, 2013, p. 386). The Jamesian framework leaves Rochat—and others—without clear mechanisms to bridge the implicit−explicit divide.

My proposed framework enables us to investigate the development of self‐representation in principled and fine‐grained ways. Various capacities—whether mirror self‐recognition, joint attention, or else—require different kinds of self‐representation, which should be understood as self‐representations of different kinds of properties. During development, the infant constructs self‐representations of increasing complexity by making explicit certain properties that were only tacitly (egocentrically) represented before. For instance, the allocentric representation of spatial properties seems to arise relatively early, around 9 months (Lew et al., 2000). This capacity is then combined with earlier egocentric representations that underpin gaze‐following (Moore, 2008) to give rise to the infant's ability to differentiate between its own and others’ attentional states at around 9−12 months (Woodward, 2003, 2005). This latter capacity requires representing attentional states allocentrically (Hauser, 2025). The development of self‐representation is thus a process of gradual enrichment, with self‐representations of different properties interlinking in complex ways.

If self‐representation develops gradually rather than emerging wholesale with *the* self‐concept at around 18 months, how should we account for the clustering of related capacities—empathy, personal pronoun use, perspective conflict detection, and more—around this same period? Something significant clearly happens at this juncture. I propose we view this as a milestone *in* self‐representation rather than *of* it. What emerges around 18 months represents a particular achievement in developing explicit self‐representation, not the birth of such representation altogether. As mentioned, this interpretation gains support from evidence that certain forms of self‐representation appear before 18 months, while others develop much later. The temporal clustering need not indicate that self‐representation itself arises at this age, but rather that specific kinds of self‐representation mature around this time. Furthermore, while various capacities do cluster around 18 months, they follow a developmental progression rather than appearing simultaneously (Michael Lewis & Ramsay, 2004). This indicates that early developing forms of self‐representation may provide the foundation for later capacities, which build upon these initial achievements by incorporating additional properties. Of course, rival explanations exist: the staggered emergence might reflect the delayed maturation of complementary cognitive mechanisms rather than the sequential development of different self‐representational capacities.

Adjudicating between these possible explanations of the 18‐month revolution requires novel empirical and theoretical research. Currently, most research merely establishes links between developing capacities without tracing their sequential emergence. While such work demonstrates that these capacities develop in tandem, it cannot establish which capacities precede others. Future longitudinal studies should, therefore, map a precise timeline of when capacities emerge and how they relate to each other. At a theoretical level, we need a clearer understanding of which properties must be self‐represented to generate specific behavioral and cognitive capacities. If my account proves correct, we should observe that capacities requiring simpler self‐representations emerge before those demanding more sophisticated ones (setting aside complementary cognitive mechanisms). We should also identify additional behaviors enabled by particular forms of self‐representation, expecting these to emerge around the same time. Crucially, on the present proposal, the later emergence of certain capacities should not result solely from complementary factors unrelated to self‐representation but rather be linked to richer coordinated allocentric representations. Additionally, we should expect these allocentric representations to be preceded by related egocentric representations with their specific cognitive and behavioral limitations.

Several important questions about self‐representation remain unexplored in this paper. While I have argued that different properties can be self‐represented, I have not examined how representations of various properties become integrated. Instantiating one allocentric representation of spatial properties and another of temporal properties differs fundamentally from representing oneself as a unified spatio‐temporal entity. Understanding when and how this integration occurs—as well as integration's limits (see Clark, 2007; Dalgleish & Power, 2004; Ismael, 2008; Lambie & Marcel, 2002)—should be a central focus of future research.

Moreover, self‐representations may initially serve only narrow, context‐specific functions before developing into more general conceptual tools (Evans, 1982). Consider allocentric spatial representation: it might first emerge purely for locomotion, only later expanding to support judgments about whether, for instance, another person could hear us from our current position. This progression from specialized to conceptual usage represents another dimension that warrants systematic investigation.

Overall, my account suggests that adult human beings are distinguished from other agents by having strongly integrated and high‐dimensional self‐representations. Our self*‐tokens ascribe numerous properties: in addition to spatial, temporal, bodily, and mental properties, we also self‐represent social properties, character traits, narrative links between events, and more. While debates about our self‐representations’ integration continue, my account shows that the difference between adult and infant—and human and animal—self‐representation is one of degree.

## 8 Conclusion

I have suggested that some organisms use coordinated allocentric representations to navigate space, keep track of temporal relations, control their bodily movements, and monitor what other people know about the world. These representations differentiate between self‐attributed and other‐attributed properties and contain a self*‐token that coordinates with sensorimotor information to directly link representation with behavior and sensation. This account also explains why certain self‐attributed properties are immune to error through misidentification, while others are not.

Systems realizing coordinated allocentric representations exemplify minimal self‐representation. As we have seen, young infants and a diverse roster of nonhuman animals can realize representations of this type, making self‐representation more common than many might have believed. Through the integration of these minimal self‐representations across various properties, more substantive forms emerge.

## Funding

This research has been supported by the Swiss National Research Foundation (grant number P500PH_202829 / 1).

## Conflicts of interest

The author hereby declares not to have any conflicts of interest (financial or nonfinancial) regarding the present research.

## Notes

1 Note that I have simplified the illustration of egocentric representation for ease of exposition. The illustrated representation is egocentric only for distance (relative to the representer's position), while bearing is already in an allocentric format (relative to a north‐south vertical).

2 What I, following Dennett (1982), call *tacit* representation is sometimes called *implicit* representation in the philosophical and psychological literature. Additionally, in the psychological literature, “implicit” and “explicit” often denote conscious and unconscious or automatic and nonautomatic processes. This is a different distinction.

3 Cognitive maps do not need to represent distances straightforwardly. As research on *predictive maps* argues, such maps might instead represent the probabilities associated with getting from one place to another (Stachenfeld, Botvinick, & Gershman, 2017). I believe such maps to be compatible with my proposal, but I cannot argue for this claim here.

4 My use of the term “coordination” should not be conflated with Grush's (2000). What I call coordination is only one variant of Grush's more general kind.

5 Some philosophers might argue that self‐representation actually combines allocentric and egocentric elements, since it requires a self‐token that arguably has egocentric content. I believe this is a terminological matter that does not affect the core of my proposal.

6 While I discussed path integration earlier in terms of egocentric representation, the process may also operate in an allocentric framework (see Charles R. Gallistel, 2008).

7 Byrne, Becker, and Burgess (2007) have recently developed a related account of *human* spatial cognition. The human case proves more complex; however, since human spatial cognition can also operate through top‐down imaginative processes (see also Klatzky, 1998).

8 A simpler explanation might be that *S1* takes on a negative valence after being paired with the footshock (*US*), and later exposure to *S2* causes fear through expectation of *S1* rather than *US*. Arcediano, Escobar, and Miller (2003) addressed this possibility with an alternative version of their experiment.

9 Forward and inverse models only explicitly differentiate between possible bodily states if *articulated* (see Grush, 2004). Such articulation is likely the case. See Wolpert and Ghahramani (2000) and Grush (2004).

10 Mirror neurons activate body representations in babies (and adults) when observing others’ actions (Lepage & Théoret, 2007), and this has been taken to suggest that infant imitation occurs because they fail to differentiate between perceived and own target states. However, the infant still needs to use some token as a self‐token, and this token cannot be what is confused with the perceived state. If she confused the other's state with her own, she would experience herself as *already* embodying the target state, making imitation unnecessary.

11 See Clayton, Dally, and Emery (2007) for details on how the experiment excluded the possibility that cache protection behavior is cued by the observer's behavior.

12 Registrations do not exemplify beliefs’ full functional profile (Butterfill & Apperly, 2013). These details are not relevant to my argument.

13 Cappelen and Dever (2014) disagree, arguing that “there is no such thing as essential indexicality, irreducibly *de se* attitudes, or self‐locating attitudes” (p. 3). Millikan (1990) argues that the relevant mental tokens *are* essential, but not indexical. I am sympathetic to this latter view, but cannot discuss it here.

14 The literature on IEM is extensive, and I can only sketch how my view relates to it here. Nonetheless, I hope this discussion demonstrates that my proposed account has the potential to explain when and why *de se* thought is IEM.

15 While uncoordinated allocentric representation commonly occurs in cognition—either because the creature does not exemplify the relevant property or because the task does not require self‐representation—various pathological cases may stem from agents’ failure to coordinate egocentric and allocentric representations. Though I lack space for detailed discussion, these cases offer intriguing research avenues. Consider deafferentation (Gallagher & Cole, 1995), where agents appear to lose the subjective route to self‐knowledge. Here, egocentric proprioceptive signals fail to update the allocentric body model. Yet coordination is not entirely blocked: once the body's state is ascertained through vision, the allocentric model can guide action. We have, hence, partial coordination—from allocentric to egocentric formats, but not in reverse. Similarly, certain symptoms of depersonalization disorder (Medford, Sierra, Baker, & David, 2005), where patients feel disconnected from their own mental states, could also imply disturbances to coordination.

16 The psychological literature freely substitutes “self” and “self‐representation” (or “self‐knowledge”), while philosophical discourse keeps these apart. A self‐representation represents the self; the self is what gets represented—an object whose existence remains contentious among philosophers. I follow the philosophical usage here, though this choice does not affect the argument.
